# Methane mitigation is associated with reduced abundance of methanogenic and methanotrophic communities in paddy soils continuously sub-irrigated with treated wastewater

**DOI:** 10.1038/s41598-021-86925-5

**Published:** 2021-04-01

**Authors:** Luc Duc Phung, Masaaki Miyazawa, Dung Viet Pham, Masateru Nishiyama, Shuhei Masuda, Fumiaki Takakai, Toru Watanabe

**Affiliations:** 1 United Graduate School of Agricultural Sciences, Iwate University, 3-18-8 Ueda, Morioka, Iwate 020-8550 Japan; 2 Faculty of Agriculture, Yamagata University, 1-23 Wakaba-machi, Tsuruoka, Yamagata 997-8555 Japan; 3 Department of Civil and Environmental Engineering, National Institute of Technology, Akita College, 1-1 Bunkyo-cho, Iijima, Akita 011-8555 Japan; 4 Faculty of Bioresource Sciences, Akita Prefectural University, 241-438 Aza Kaidobata-Nishi, Shimoshinjo Nakano, Akita, 010-0195 Japan

**Keywords:** Environmental impact, Agroecology, Wetlands ecology, Soil microbiology

## Abstract

Herein, we examined emissions of CH_4_ and the community structures of methanogenic archaea and methanotrophic bacteria in paddy soils subjected to a novel irrigation system, namely continuous sub-irrigation with treated wastewater (TWW). This system has recently been developed by our group to effectively reuse TWW for the cultivation of protein-rich rice. The results showed that, despite not using mineral fertilisers, the wastewater reuse system produced a rice yield comparable to that of a conventional cultivation practice and reduced CH_4_ emissions from paddy fields by 80%. Continuous sub-irrigation with TWW significantly inhibited the growth of methanogens in the lower soil layer during the reproductive stage of rice plants, which was strongly consistent with the effective CH_4_ mitigation, resulting in a vast reduction in the abundance of methanotrophs in the upper soil layer. The compositions of the examined microbial communities were not particularly affected by the studied cultivation practices. Overall, this study demonstrated that continuous sub-irrigation with TWW was an effective method to produce high rice yield and simultaneously reduce CH_4_ emissions from paddy fields, and it also highlighted the potential underlying microbial mechanisms of the greenhouse gas mitigation.

## Introduction

Methane (CH_4_) is one of the major greenhouse gasses (GHGs) associated with human activity and has contributed significantly to global warming^1^. Rice cultivation is one of the largest anthropogenic sources of CH_4_, accounting for approximately 20% of its global emissions from agriculture^2^. The growing demand for rice to feed the ever-increasing global population has resulted in a continuous increase in rice production, which is most likely intensified CH_4_ emissions. In addition, increasing rice production inevitably requires higher inputs of mineral fertilisers into paddy soils, which is considered to be the key driver contributing to the overall C footprint of paddy rice farming^3,4^. Furthermore, the rapidly rising amount of fertilisers used in rice cultivation causes other problems, such as water pollution, soil acidification, soil mineral depletion, and rising cost of production^5^. Thus, there is a critical need for sustainable rice cultivation systems that effectively mitigate CH_4_ emissions and reduce the use of mineral fertilisers while maintaining or improving rice productivity.

We have recently introduced an innovative irrigation system for the cultivation of paddy rice^6^, namely continuous sub-irrigation with treated wastewater (hereinafter, referred to as CSI). The objective of this system is to promote the recycling of nutrients from municipal wastewater treatment plants (WWTPs) in a cost-effective manner and the production of high yielding protein-rich rice without the use of mineral fertilisers^6–8^. Importantly, although it was previously claimed that irrigation with wastewater would significantly increase CH_4_ emissions due to the high availability of organic C in irrigation wastewater^9^, our recent study found that CSI could markedly reduce CH_4_ emissions by up to 84% compared with conventional rice cultivation^6^. However, the underlying mechanisms for this mitigation have not yet been thoroughly investigated.

In general, CH_4_ emissions from rice paddy fields are strongly affected by microbial activity in soils. CH_4_ is produced by methanogenic archaea (methanogens) under anaerobic conditions in the rhizosphere; however, in these conditions, a portion of the produced gas is oxidised by methanotrophic bacteria (methanotrophs) before it is released into the atmosphere^10^. In addition, since the metabolic activity of soil microorganisms is particularly stimulated by irrigation with wastewater^11^, this wastewater reuse practice has been hypothesized to change soil microbial community structures and subsequently influence the emission patterns of GHGs from paddy soils^9^. The emission of CH_4_ in relation to changes in soil microbial communities caused by various agricultural management practices, particularly chemical and organic fertilisation, has been well documented^12–15^. However, to the best of our knowledge, no studies have been conducted on soil microbial communities associated with CH_4_ emissions from rice paddy fields irrigated with wastewater, especially those using CSI, in which TWW acts as the sole source of both irrigation and fertilisation for rice plants^6^.

Understanding how CH_4_ emissions are associated with the relevant microbial communities in the newly-introduced rice cultivation system remains a fundamental question that needs to be addressed. Therefore, the present study acts as a follow-up research of our previous work^6^ aiming (1) to verify the impacts of CSI on CH_4_ emissions in comparison with that of a conventional rice cultivation, and (2) to investigate the community structures of methanogens and methanotrophs in paddy soils under the two cultivation systems and the possible dominant microbial mechanisms involved in the CSI-induced influences on CH_4_ emissions.

## Results

### Seasonal dynamics of CH_4_ fluxes

**Figure 1:**
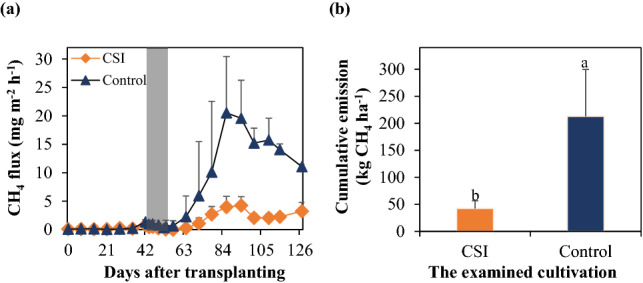
Seasonal dynamics of CH_4_ fluxes (**a**) and cumulative emission of CH_4_ (**b**) from the paddy fields as influenced by the examined treatments. The fluxes are the means of the values measured within each treatment (n = 3). The grey belt indicates the time of the MSD period, while the error bars denote the standard deviation of the means. Different letters above the error bars identify a significant difference between the two treatments (*p* < 0.05).

Daily fluxes of CH_4_ estimated from CSI system and a conventional cultivation practice (Control) had similar patterns (Fig. 1a). CH_4_ emissions were not notably affected by the two treatments during the vegetative growth phase from 0 to 56 days after transplanting (DAT) and thereafter, substantial fluctuations were observed during the reproductive stage (64 DAT onwards).

In the early growth stage, CH_4_ emissions from the paddy fields were negligible (< 1.37 mg CH_4_ m^−2^ h^−1^), regardless of the treatments. However, the gas fluxes from both Control and CSI treatments markedly increased from the start of the booting time (63 DAT) and reached their peaks of 20.5 and 4.3 mg CH_4_ m^−2^ h^−1^ during the flowering stage (86 and 94 DAT, respectively). Thereafter, the fluxes from Control decreased sharply to their minimum (11.1 mg CH_4_ m^−2^ h^−1^) at the end of the crop season, whereas those from CSI gradually reduced and remained at considerably lower levels (2.1–3.3 mg CH_4_ m^−2^ h^−1^) during the late growing period. Overall, CSI exhibited a seasonal average flux of 1.1 ± 1.4 mg CH_4_ m^−2^ h^−1^, which was markedly lower than that of Control (5.5 ± 7.2 mg CH_4_ m^−2^ h^−1^). Consequently, CSI significantly reduced the cumulative emissions of CH_4_ compared with Control (212.8 kg ha^−1^) by 80% (*p* < 0.05; Fig. 1b).

### Rice yield and yield-scaled emissions of CH_4_

**Figure 2:**
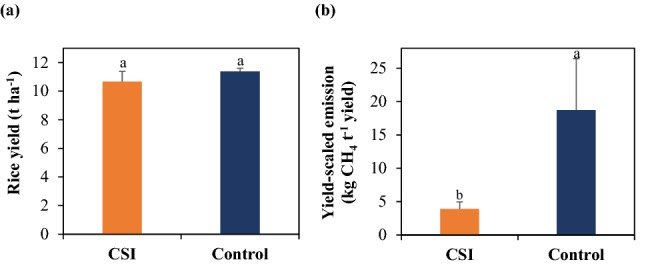
Rice yield (**a**) and yield-scaled emission of CH_4_ (**b**) as influenced by the examined treatments. The data are the means of the values measured within each treatment (n = 3). Error bars denote the standard deviation of the means. Different letters above the error bars identify a significant difference between the two treatments (*p* < 0.05).

**Figure 3:**
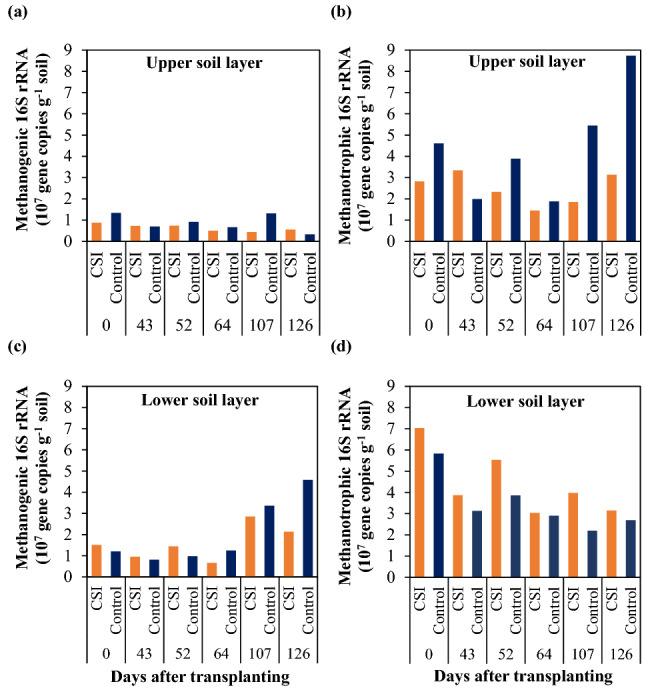
Abundance of methanogenic archaea (**a**,**c**) and methanotrophic bacteria (**b**,**d**) in the upper and lower layers of the paddy soils, respectively, under the examined treatments.

Figure 2 presents the brown rice yield and yield-scaled emissions of CH_4_ from the examined treatments. CSI produced a rice yield of 10.7 t ha^−1^, which was comparable to that (11.4 t ha^−1^) recorded in Control (*p* > 0.05; Fig. 3a), while the trend in yield-scaled CH_4_ emissions between the two treatments was similar to that observed in the cumulative emissions of the gas. Particularly, CSI produced 3.9 kg CH_4_ t^−1^ of yield, which was 79% lower than that of Control (18.7 kg CH_4_ t^−1^ yield; *p* < 0.05; Fig. 3b).

### Abundance of methanogens and methanotrophs in paddy soils

The abundance of methanogenic and methanotrophic communities varied considerably during the entire crop season (Fig. 3). Consistently in both treatments, the abundance of methanogenic archaea was markedly lower than that of methanotrophic bacteria, especially in the upper soil layer, where the loads of methanogens (3.3 × 10^6^–1.3 × 10^7^ gene copies g^−1^ soil) were vastly smaller than those of methanotrophs (1.5 × 10^7^–8.7 × 10^7^ gene copies g^−1^ soil). In addition, the target microbes generally had higher abundance in the lower soil layer than in the upper layer, regardless of the examined treatments.

Across six sampling time-points (0, 43, 52, 64, 107, and 126 DAT), CSI treatment tended to have a lower abundance of methanogens than Control. Particularly, the copy numbers of methanogenic archaeal 16S rRNA genes recorded in the upper and lower soil layers with CSI treatment, averaged 6.4 × 10^6^ and 1.6 × 10^7^ gene copies g^−1^ soil, respectively, which were 27 and 20% lower than those (8.8 × 10^6^ and 2.0 × 10^7^ gene copies g^−1^ soil) observed with Control (Fig. 3a,c). It was noteworthy that the gap in the numbers of methanogenic archaea between CSI and Control treatments were more prominent during the reproductive stage (from 64 DAT onwards, Fig. 3a,c). For methanotrophic bacteria, however, CSI immensely reduced the average abundance of methanotrophs (2.5 × 10^7^ gene copies g^−1^ soil) by more than 43% relative to Control (4.4 × 10^7^ gene copies g^−1^ soil) in the upper soil layer (Fig. 3b). In contrast, the methanotrophic community in the lower soil layer with CSI treatment (4.4 × 10^7^ gene copies g^−1^ soil) was 18% more abundant than that (3.4 × 10^7^ gene copies g^−1^ soil) with the Control (Fig. 3d). Furthermore, the pairwise correlation analyses (Table 1) showed that CH_4_ fluxes had a significantly positive relationship with the abundance of methanotrophic bacteria (r = 0.7, *p* < 0.05) while possessing no significant correlation with the methanogenic community in the upper soil layer. In the lower layer of paddy soils, however, the gas fluxes had no significant relationship with the methanotrophic community, but strongly and positively correlated (r = 0.82, *p* < 0.01) with the abundance of methanogenic community (Table 1). Overall, there were noticeable dynamic changes in the abundance of the soil microbial communities with CSI treatment in contrast to Control throughout the crop season.

**Table 1:** Correlation coefficients between CH_4_ fluxes and methanogenic archaea and methanotrophic bacteria in the upper and lower soil layers across the crop season.

Parameter	CH_4_ fluxes	M1	M2	M3	M4
CH_4_ fluxes	1	0.15	0.70*	0.82**	− 0.58
M1	0.15	1	0.13	− 0.19	0.29
M2	0.70*	0.13	1	0.75**	− 0.21
M3	0.82**	− 0.19	0.75**	1	− 0.33
M4	− 0.58	0.29	− 0.21	− 0.33	1

M1 and M2, Methanogens and Methanotrophs in the upper soil layer, respectively; M3 and M4, Methanogens and Methanotrophs in the lower soil layer, respectively.

**Correlation is significant at *p* < 0.01.

*Correlation is significant at *p* < 0.05.

### Community composition of methanogens and methanotrophs in paddy soils

**Figure 4:**
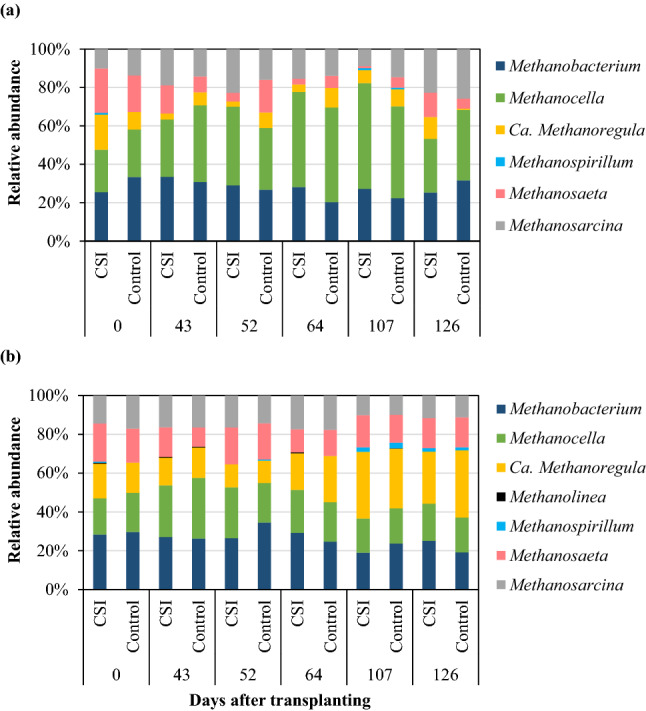
Taxonomic composition of the methanogenic archaea in the upper (**a**) and lower layers (**b**) of the paddy soils under the examined treatments.

The methanogenic community consisted of 7 archaeal genera in all soil samples, as shown in Fig. 4, in which *Methanolinea* was irregularly found only in the lower soil layer. In the upper soil layer with CSI treatment, *Methanocella* and *Methanobacterium* were the predominant genera, accounting for 37% and 28% of the community, respectively, followed by *Methanosarcina* (16%), *Methanosaeta* (10%), and *Ca. Methanoregula* (8%). *Methanospirillum* had the lowest abundance, and it occasionally found at negligible loads (approximately 1%, Fig. 4a). This trend, with similar relative abundance of the genera, was also found with Control in the same soil layer, with the following genera (arranged in the order of dominance): *Methanocella* (38%), *Methanobacterium* (27%), *Methanosarcina* (16%), *Methanosaeta* (10%), *Ca. Methanoregula* (7%), and *Methanospirillum* (< 1%). Similarly, there was a consistency in the relative abundance of methanogenic genera between CSI and Control treatments in the lower soil layer (Fig. 4b). The three most abundant methanogens were *Methanobacterium*, *Ca. Methanoregula*, and *Methanocella* (approximately 21–26%), followed by *Methanosaeta* and *Methanosarcina* (14–16%), while the rest of the genera that were accounted for had negligible contributions (< 1%).

**Figure 5:**
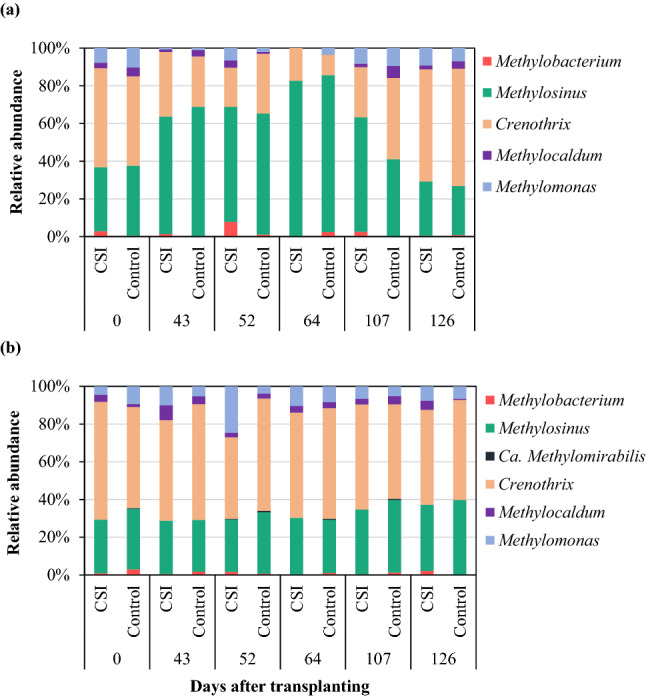
Taxonomic composition of the methanotrophic bacteria in the upper (**a**) and lower layers (**b**) of the paddy soils under the examined treatments.

The methanotrophic community consisted of 6 bacterial genera as presented in Fig. 5, in which *Ca. Methylomirabilis* was detected only in the lower soil layer. Consistently in both the upper and lower soil layers, the most dominant methanotrophic bacteria were *Methylosinus* and *Crenothrix,* regardless of the treatments. In the upper soil layer, the copy numbers of *Methylosinus* averaged 55% and 53% for CSI and Control treatments, respectively, whereas *Crenothrix* had average copy numbers of 35% and 37% for the two treatments, respectively (Fig. 5a). In contrast to the upper soil layer, the growth of *Crenothrix* was stimulated in the lower layer, accounting for 54% and 56% of the methanotrophic communities treated with CSI and Control, respectively. The relative abundance of *Methylosinus* treated with CSI (31%) was comparable to that of the Control (33%; Fig. 5b). The rest of the methanotrophic genera had uniform contributions at minor proportions (< 10%) to the total methanotrophic bacterial communities, regardless of the examined cultivation practices. Thus, while no notable distinction in the microbial compositions was observed between CSI and Control, there was an apparent variation in the compositions of the identified genera between the upper and lower soil layers in both cultivation systems.

## Discussion

High levels of fertilisation are expected to result in high yields of forage rice^16^. The rice plants used in this study were cv. Bekoaoba, which is a local forage rice that generally requires a high dose of mineral fertiliser supplementation. However, in spite of zero use of mineral fertilisers, CSI treatment still produced a comparable yield that were much higher than the target of 8 t ha^−1^ for the variety Bekoaoba^17^ and the average yield (7.3 t ha^−1^) obtained from conventional paddy fields in the same region^7^. The high yield obtained from CSI treatment was primarily attributed to the effective assimilation of the plant nutrients, especially the high concentrations of N (Table 2) derived from the irrigation TWW during the entire crop season^6^. This finding was in agreement with that recorded in our previous investigations on the paddy fields employing the same wastewater reuse system^6–8^. In addition, the effective mitigation of CH_4_ emissions under CSI was consistent with our preliminary findings^6^, in which CH_4_ emissions from a conventional cultivation practice were also reduced by 70–84%, with the use of CSI. Therefore, this follow-up study has verified the appealing advantages of CSI in rice paddy farming, i.e., producing high rice yields with reduced emissions of CH_4_ and simultaneously eliminating the use of mineral fertilisers.

**Table 2:** Properties of TWW used for continuous sub-irrigation during the crop season.

Parameter	Unit	May	June	July	August	September	Mean
pH		7.2	7.1	6.5	7.3	6.5	6.9
EC	dS m^−1^	0.7	0.6	0.6	0.6	0.6	0.6
DO	mg L^−1^	1.4	1.5	2.5	2.2	2.2	1.9
TOC	mg L^−1^	6.9	4.3	6.1	6.1	5.8	5.8
N	mg L^−1^	32	28	25	27	28	28
P	mg L^−1^	0.2	0.1	0.1	0.3	0.3	0.2
K	mg L^−1^	17.1	17.1	18.2	18.6	18.5	17.9

Rice paddy fields are among the most common habitats suitable for the growth and development of methanogens that are obligate anaerobic microbes producing CH_4_ as an essential component of their energy metabolism^10^. The methanogenic genera identified in this study were those commonly found in rice paddy fields, sewage sludge, and freshwater sediments^18^. In contrast to methanogens, methanotrophs are classified as aerobic bacteria frequently found at the oxic-anoxic interfaces of specific environments like wetlands, aquatic sediments, and rice paddy fields, where they can oxidize 10–90% of the CH_4_ produced by methanogens in lower anoxic zones before the gas reaches the atmosphere^10,19^. The methanogenic and methanotrophic genera identified in the present study have been clearly described elsewhere^10,18–20^. The similarity on the relative abundance of these microbes under CSI and Control treatments was probably a result of the predominant flooding condition consistently in both cultivation systems, which thereby determined the soil microbial compositions in a similar fashion, despite their differences in fertilisation. This was likely due to the fact that flooding had a greater effect than nutrient loading on altering both the composition as well as the functional components of soil microbial communities^21^.

The explicit dissimilarity in the structures of the examined communities between the upper and lower soil layers was supported by the non-uniformed relationship between the CH_4_ fluxes and the abundance of methanogenic and methanotrophic communities in the two soil layers. In particular, the significantly positive correlation between the gas fluxes and the abundance of methanotrophic bacteria in the upper soil layer was most likely owing to the shallow oxic surface of the flooded soil in the upper layer that is suitable for the growth of aerobic methanotrophs but not for anaerobic methanogens^10^. This also explains the lower abundance of methanogens in the upper soil layer compared with the lower layer, regardless of the treatments. On the other hand, the strong and positive relationship between the CH_4_ fluxes and the abundance of methanogens in the lower soil layer was primarily attributed to the anoxic and reduced conditions in the deep soil layer, which is suitable for the growth and development of anaerobic methanogens, but inhibits the aerobic methanotrophic community^10^. Our results were in line with a previous report demonstrating that CH_4_ emissions were positively correlated with the transcripts of the *mcr*A and *pmo*A genes^22^, which were used as phylogenetic markers and a mean to calculate the abundance of methanogens and methanotrophs, respectively^23^. However, these results were in contrast with another study^13^, which claimed that CH_4_ fluxes were positively related to the *mcr*A gene copy numbers but negatively related to the *pmo*A gene copy numbers in paddy soils.

**Figure 6:**
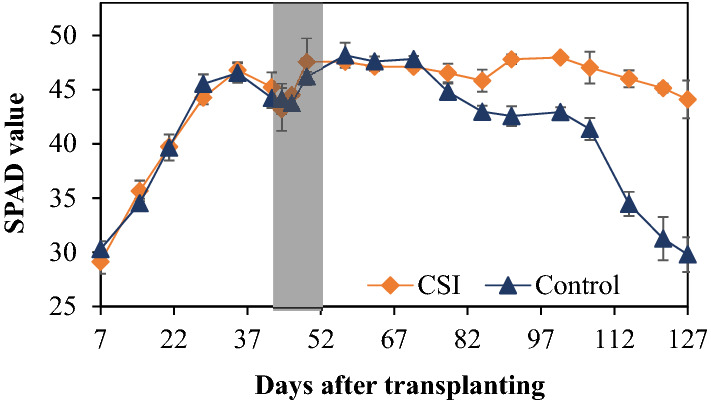
Soil plant analysis development (SPAD) value of the rice leaves under the examined treatments during the crop season. The SPAD values are the means within each treatment (n = 3). The grey belt indicates the time of the MSD period, while the error bars denote the standard deviation of the means.

In the lower soil layer, the higher copy number of methanogenic 16S rRNA genes with CSI treatment during the vegetative growth period (0–52 DAT) was likely attributed to the higher input of total organic carbon (TOC) in TWW (Table 2), compared to the tap water used in Control. However, this difference did not translate into a notable variation in CH_4_ fluxes, which were comparable between CSI (0.09, 0.44, and − 0.004 mg CH_4_ m^−2^ h^−1^) and Control treatments (0.09, 1.04, and 0.37 mg CH_4_ m^−2^ h^−1^) across the first three times of sampling (0, 44, and 52 DAT, respectively). These CH_4_ fluxes were tremendously lower than those measured during the reproductive stage (around 64 DAT onwards, Fig. 1a) regardless of the treatments, which was in line with the lower and higher abundance of methanogens in the former and later growth periods, respectively. Regardless of the examined treatments, the lower CH_4_ emissions and the lower abundance of methanogens in the deep soil during the vegetative stage relative to those in the reproductive period were likely a result of lower availability of C substances in the soils. It was demonstrated that almost 100% of the CH_4_ produced in the early growth period were originated from rice straw and soil organic matter (SOM)^24^. In this microcosm experiment, the soils were homogenized and all plant residues were carefully removed before potting, thus leaving a relatively low content of organic matter in the soils. Later in the crop season, however, photosynthesis became a more important source for CH_4_ production. The high peaks recorded for CH_4_ fluxes from both CSI and Control treatments during the grain filling period (around 86 DAT onwards) were most likely attributed to high availability of C substances in the paddy soils. These C substances primarily originated from root exudates and decaying root debris^25^, which were at the highest rates during the flowering period (about 80 DAT) when compared to the other growth stages of the rice plants^26^. Since the photosynthetic products derived from root exudates were generally the main C substrates contributing to 65–70% of total CH_4_ emissions during this period^24^, higher root exudation in the flowering time could greatly stimulate CH_4_ fluxes during the following growth stage^26^. From 64 DAT onwards, the lower abundance of methanogens with CSI treatment relative to Control was likely a result of lower amounts of C substances available for the microbial metabolism. Consistently with our previous findings^6^, CSI was found to postpone the physiological senescence of rice plants as having higher levels of leaf greenness^27^, which was expressed as Soil Plant Analysis Development-SPAD value (Fig. 6), thereby maintaining effective photosynthetic activities of the rice plants when the maturity approached. As a result, CSI could likely reduce the rate of root exudation and decaying in the rhizosphere, which could inhibit CH_4_ production in the soils by limiting C substrates of the methanogenesis process^28,29^.

In the upper soil layer, on the other hand, the higher abundance of methanotrophs under Control treatment was probably owing to the larger amounts of CH_4_ produced in the deep soil compared to CSI treatment. Larger amounts of CH_4_ could be then diffused upwards, resulting in the stimulated methanotrophy process in the upper soil layer in Control. Overall, the CSI-induced reduction of CH_4_ was attributed to the inhibition of methanogenic community in the lower soil layer, while the reduced abundance of methanotrophic community under CSI was probably due to the lower CH_4_ production in the deep soil layer and subsequently the lower CH_4_ diffusion into the upper layer of the paddy soils.

In conclusion, we have demonstrated an effective method of wastewater reuse to produce high rice yields and reduce CH_4_ emissions while diminishing the dependence of paddy rice farming on mineral fertilisers and fresh water from conventional sources. The effective mitigation of CH_4_ emissions from rice paddy fields using CSI has been verified herein and the possible underlying mechanism of such the mitigation has been identified. Given that the patterns in CH_4_ emissions from rice paddy fields are associated with real-time variation in other soil chemical properties during the entire crop season, such as soil pH, dissolved oxygen (DO), porosity, N status, soil organic acids, and SOM^15,23,30^, follow-up studies are highly recommended to focus on GHG emissions linking with dynamic changes in soil microbial communities as well as other soil physicochemical properties in real-scale rice fields using CSI.

## Methods

### Experimental design and crop establishment

A microcosm experiment was conducted at Yamagata University, Tsuruoka City, Japan, from May to October 2019, with six growth containers (36 cm in height, 30 cm in width, 60 cm in length) to simulate paddy fields of 0.18 m^2^ in area (see Supplementary Fig. S1). The experiment was laid out in a completely randomized design with three replications of two treatments: (1) rice cropping under CSI and (2) conventional rice cultivation fertilized with mineral fertilisers and irrigated with tap water (Control).

Each container was filled with 32 kg of a paddy soil collected from an experimental field in the university farm and transplanted with four hills of 30-day-old seedlings (*Oryza sativa* L., cv. Bekoaoba) on 27th May 2019. The experiment was performed in accordance with relevant guidelines and regulations for research involving plants. The experimental soil was classified as loamy soil (air-dried, 20% moisture) with the following basic properties: pH (H_2_O) of 5.78, electrical conductivity (EC) of 0.09 dS m^−1^, SOM of 4.9%, and a total N, P, and K of 1.46, 0.88, and 3.17 g kg^−1^, respectively. The TWW used in the CSI system was collected from a local WWTP and monitored weekly for its basic properties (Table 2) following our previous studies^6,7^. In brief, pH, EC, and DO of water samples were measured on-site using pH/conductivity and DO portable meters (D-54 and OM-51, HORIBA, Ltd., Kyoto, Japan), whereas TOC and total N were analyzed using a TOC analyzer (TOC-VCSV, Shimadzu Corp., Kyoto, Japan) attached to a total N measuring unit (TNM-1, Shimadzu Corp., Kyoto, Japan). After a standard acid-digestion of water samples^6^, the concentration of P was measured using a portable colorimeter (DR/890, HATCH, USA), and the concentration of K was measured using an inductively coupled plasma mass spectrometry (ICP-MS ELAN DRCII, PerkinElmer Japan Co., Ltd.). The tap water used in this study was also tested on a regular basis and found to be stable throughout the crop season, with the following properties: pH of 7.8, EC of 0.095 dS m^−1^, DO, TOC, N, and P of 6.85, 0.49, 0.06, and 0.07 mg L^−1^, respectively, with K being below the ICP-MS detection limit (< 0.3 mg L^−1^).

The CSI system has been described thoroughly in our previous study^6^. Briefly, TWW was supplied to the experimental containers through underground perforated pipes from which TWW infiltrated the soil layers and eventually overflowed out of the simulated paddy fields (see Supplementary Fig. S1). The optimal irrigation rate suggested in our prior investigation was adopted as follows: a flow rate of 25 L m^−2^ day^−1^ was implemented during the active tillering and reproductive periods, and a lower rate of 8.3 L m^−2^ day^−1^ was implemented at the early tillering and late ripening stages^6^. Irrigation with TWW was initiated 3 DAT and maintained continuously throughout the crop season. No exogenous fertiliser was applied with CSI treatment. In the Control, rice plants were conventionally supplemented with high doses of mineral fertilisers for basal (160 kg N–P_2_O_5_–K_2_O ha^−1^) and topdressing fertilisation (100 kg N–K_2_O ha^−1^), while daily irrigation was conducted by manually adding tap water to maintain 5 cm of standing water above the soil surface. Consistent with both treatments, irrigation was withheld from 43 to 52 DAT for a mid-season drainage (MSD). The rice plants were harvested on 1st October 2019 and rice yields were assessed as the weight of brown rice at 15% moisture.

### CH_4_ emission measurement

The static closed chamber method^31^ was used to sample the gas as described in our previous study^6^. Briefly, before the gas sampling, six transparent static chambers were mounted securely on the growth containers at the water-seal collars (see Supplementary Fig. S1). Gas samples were extracted from the chambers 0, 15, and 30 min after the chamber deployment and immediately transferred to the laboratory for CH_4_ measurement using a gas chromatograph (GC-2014, Shimadzu Corp., Kyoto, Japan). The gas was collected from around 10:00 a.m. to 11:00 a.m. once a week throughout the crop season, while the sampling frequency was increased to once every 2 days during the MSD period.

Daily fluxes and seasonal cumulative emissions of CH_4_ were calculated following the standard procedures as described previously^31^. In order to correlate the cumulative emission of CH_4_ to the rice production, the yield-scaled emission was calculated using the following equation:

$$ {\text{YSE}} = \frac{{{\text{CE}}}}{{\text{Y}}}, $$

where, YSE, CE and Y represents the yield-scaled emission (kg CH_4_ t^−1^ yield), the cumulative emission (kg CH_4_ ha^−1^) and the yield of brown rice (t ha^−1^), respectively.

### Soil sampling

The paddy soils were sampled at six time-points, which were representative of different soil conditions and growth stages of the rice plants during the crop season: pre-transplantation, maximum tillering, panicle initiation, booting, grain filling, and ripening stages (0, 43, 52, 64, 107, and 126 DAT, respectively). Samples were collected in each container using a soil core (1.5 cm in diameter × 15 cm in depth) from which the upper and lower layers of the paddy soil (0–1 and 12–15 cm, respectively) were collected separately. The respective soil layers from three replicates were mixed to form one composite sample of each treatment, placed in a 50 mL commercial centrifuge tube (VWR SuperClear Ultra-High Performance Freestanding Centrifuge Tubes with Flat Caps, VWR international, USA) and then transferred to the laboratory and immediately stored at − 80 °C.

### DNA extraction, PCR, and quantitative PCR assays

Soil DNA was extracted from 24 samples (2 treatments × 2 layers × 6 time points) using 0.25 g of the frozen soils as the input for the DNeasy PowerSoil Kit (QIAGEN, Hilden, Germany) following the manufacturer’s instructions. Next, DNA concentration was determined using Qubit 4 Fluorimeter (Thermo Fisher Scientific, Waltham, Massachusetts, USA) and the total DNA extracts were stored at − 80 °C until further analysis.

The 24 total DNA extracts were all used for Illumina MiSeq 16S rRNA gene sequencing. Amplicon library preparation and Illumina MiSeq sequencing were performed by Fasmac Co., Ltd. (Atsugi, Japan). The universal primers U515F (5′-ACACTCTTTCCCTACACGACGCTCTTCCGATCTGTGCCAGCMGCCGCGGTAA-3′) and U806R (5′-GTGACTGGAGTTCAGACGTGTGCTCTTCCGATCTGGACTACHVGGGTWTCTAAT-3′) were used following a previous study^32^. They targeted the V4 hypervariable regions of the archaeal and bacterial 16S rRNA genes and were used for the first 16S rRNA gene amplification^33^. Operational taxonomic units (OTUs) were defined at the sequence similarity level of 97% and a representative sequence from each OTU was assigned to a taxonomic identity using the Quantitative Insights into Microbial Ecology (QIIME) software package^34^. Compositions of the archaeal and bacterial communities (at the genus level) in the soil samples were determined by classifying the taxa of each OTU using the Greengenes database at Fasmac Co., Ltd., Japan.

In order to evaluate the total load of archaeal and bacterial communities in the soil samples, two real-time PCR (qPCR) assays targeting the 16S rRNA genes of the archaeal and bacterial DNA were performed, respectively, using a CFX96 Touch Real-Time Detection System (Bio-Rad Laboratories, Inc. Hercules, CA, USA). The primers and probes used for the assays were previously developed^35,36^. The standards (10^2^–10^6^ gene copies) for the qPCR assays of archaea were prepared using the strain NBRC110930 *Haloarchaeobius iranensis*, while those for the qPCR assay of bacteria were prepared using the strain NBRC3301 *Escherichia coli* K12. The reaction conditions for the amplification of archaea were as follows: 95 °C for 10 min, followed by 40 cycles at 95 °C for 20 s and 60 °C for 1 min; in contrast, the conditions for bacterial amplification were as follows: 50 °C for 2 min and 95 °C for 10 min, followed by 50 cycles of 95 °C for 15 s and 60 °C for 1 min. The abundance of methanogenic and methanotrophic genera was calculated based on their proportions compared to the quantity of the total archaeal and bacterial communities in the soil samples.

### Statistical analysis

Differences in CH_4_ emissions between the examined treatments were evaluated using Student’s *t* tests at a significance level of 0.05. The Pearson correlation analysis was carried out between CH_4_ fluxes and the abundance of methanogenic and methanotrophic communities, using the data derived from the 6 time-points across the treatments. All statistical analyses were performed using IBM SPSS Statistics 24.0.

## Supplementary Information

Supplementary Figure S1.
