# Supplementary material for: Methane mitigation is associated with reduced abundance of methanogenic and methanotrophic communities in paddy soils continuously sub-irrigated with treated wastewater
Source: Sci Rep. 2021 Apr 1;11:7426. doi: 10.1038/s41598-021-86925-5 (PMC8016930; doi:10.1038/s41598-021-86925-5)
Supplement: Supplementary file 1 — Supplementary Figure S1. [file 41598_2021_86925_MOESM1_ESM.docx]

**Methane Mitigation is Associated with Reduced Abundance of Methanogenic and Methanotrophic Communities in Paddy Soils Continuously Sub-irrigated with Treated Wastewater**

**Luc Duc Phung^1,*^, Masaaki Miyazawa^2^, Dung Viet Pham^2^, Masateru Nishiyama^2^, Shuhei Masuda^3^, Fumiaki Takakai^4^, and Toru Watanabe^2,*^**

^1^ United Graduate School of Agricultural Sciences, Iwate University, 3-18-8 Ueda, Morioka, Iwate 020-8550, Japan

^2^ Faculty of Agriculture, Yamagata University, 1-23 Wakaba-machi, Tsuruoka, Yamagata 997-8555, Japan

^3^ Department of Civil and Environmental Engineering, National Institute of Technology, Akita College, 1-1 Bunkyo-cho, Iijima, Akita 011-8555, Japan.

^4^ Faculty of Bioresource Sciences, Akita Prefectural University, 241-438 Aza Kaidobata-Nishi, Shimoshinjo Nakano, Akita 010-0195, Japan.

* Corresponding authors: Luc Duc Phung ([phungducluc0305@gmail.com](mailto:phungducluc0305@gmail.com)), Toru Watanabe ([to-ru@tds1.tr.yamagata-u.ac.jp](mailto:to-ru@tds1.tr.yamagata-u.ac.jp))

**Supplementary Information**

| **(a)**   | **(b)**  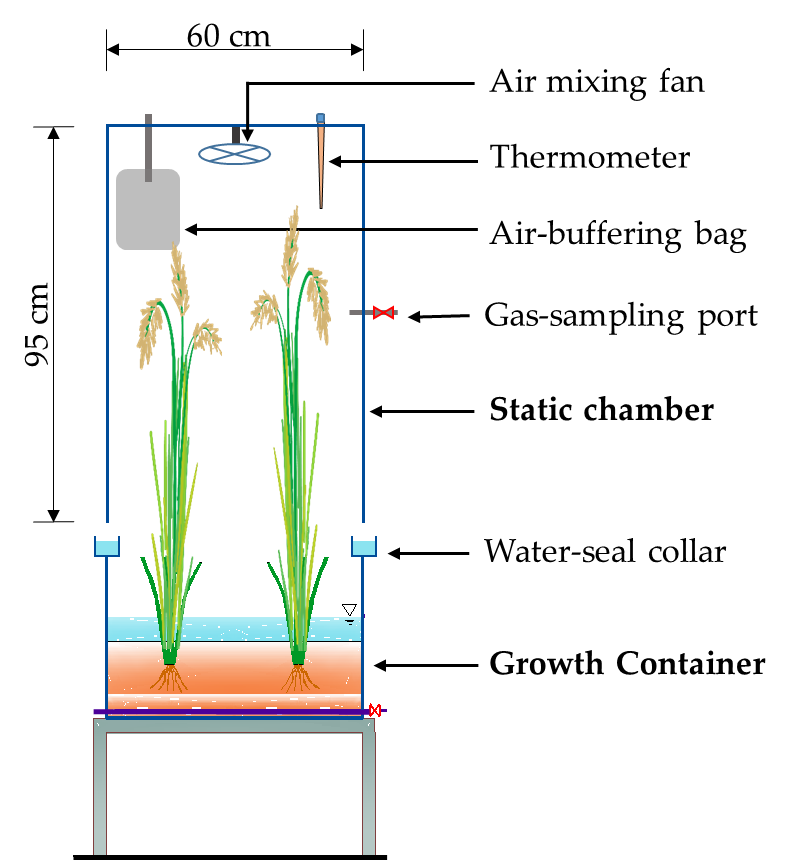 |
| --- | --- |

**Figure S1.** Schematic illustration of the growth container equipped with the continuous sub-irrigation system (**a**) and the static chamber used for gas sampling (**b**).
